# HIV Transmembrane Glycoprotein Conserved Domains and Genetic Markers Across HIV-1 and HIV-2 Variants

**DOI:** 10.3389/fmicb.2022.855232

**Published:** 2022-05-27

**Authors:** Ana Valadés-Alcaraz, Roberto Reinosa, África Holguín

**Affiliations:** HIV-1 Molecular Epidemiology Laboratory, Instituto Ramón y Cajal de Investigación Sanitaria (IRYCIS), Microbiology Department, Hospital Universitario Ramón y Cajal, CIBER en Epidemiología y Salud Pública (CIBERESP), Red en Investigación Translacional en Infecciones Pediátricas (RITIP), Madrid, Spain

**Keywords:** HIV, transmembrane glycoprotein gp41, transmembrane glycoprotein gp36, conservation, variants, antibody binding domains

## Abstract

HIV envelope transmembrane glycoproteins gp41 (HIV-1) and gp36 (HIV-2) present high variability and play a key role in the HIV-host cell membrane's fusion, as a target for human broadly neutralizing antibodies (bnAbs) and drugs. Thus, a better knowledge of amino acid (aa) conservation across structural domains and HIV variants can help to identify conserved targets to direct new therapeutic and diagnostic strategies. All available gp41/gp36 nucleotide sequences were downloaded from Los Alamos National Laboratory (LANL) HIV Sequence Database, selecting 17,078 sequences ascribed to HIV-1 and HIV-2 variants with ≥3 sequences. After aligning and translating into aa with MEGAv6.0, an in-house bioinformatics program (EpiMolBio) was used to identify the most conserved aa and the aa changes that were specific for each variant (V-markers) *vs*. HXB2/BEN (HIV-1/HIV-2) reference sequence. We analyzed the presence of specific aa changes among V-markers affecting infectivity, gp41 structure, function, or resistance to the enfuvirtide viral fusion inhibitor (T-20). We also inferred the consensus sequences per HIV variant, describing in each HIV-1 group (M, N, O, P) the conservation level along the complete gp41 per structural domain and locating in each binding site the anti-gp41 human Abs (bnAbs and non bnAbs) described in LANL. We found 38.3/59.7% highly conserved aa present in ≥90% of the 16,803/275 gp41/gp36 sequences ascribed to 105/3 HIV-1/HIV-2 variants, with 9/12.6% of them showing complete conservation across LANL sequences. The fusion peptide, its proximal region, the N-heptad repeat, and the membrane-proximal external region were the gp41 domains with ≥84% of conserved aa in the HIV-1 consensus sequence, the target of most Abs. No natural major resistance mutations to T-20 were observed. Our results show, for the first time, a complete conservation study of gp41/gp36 per variant in the largest panel of HIV variants analyzed to date, providing useful information for a more rational design of drugs, vaccines, and molecular detection tests targeting the HIV transmembrane glycoprotein.

## Introduction

**Figure 1:**
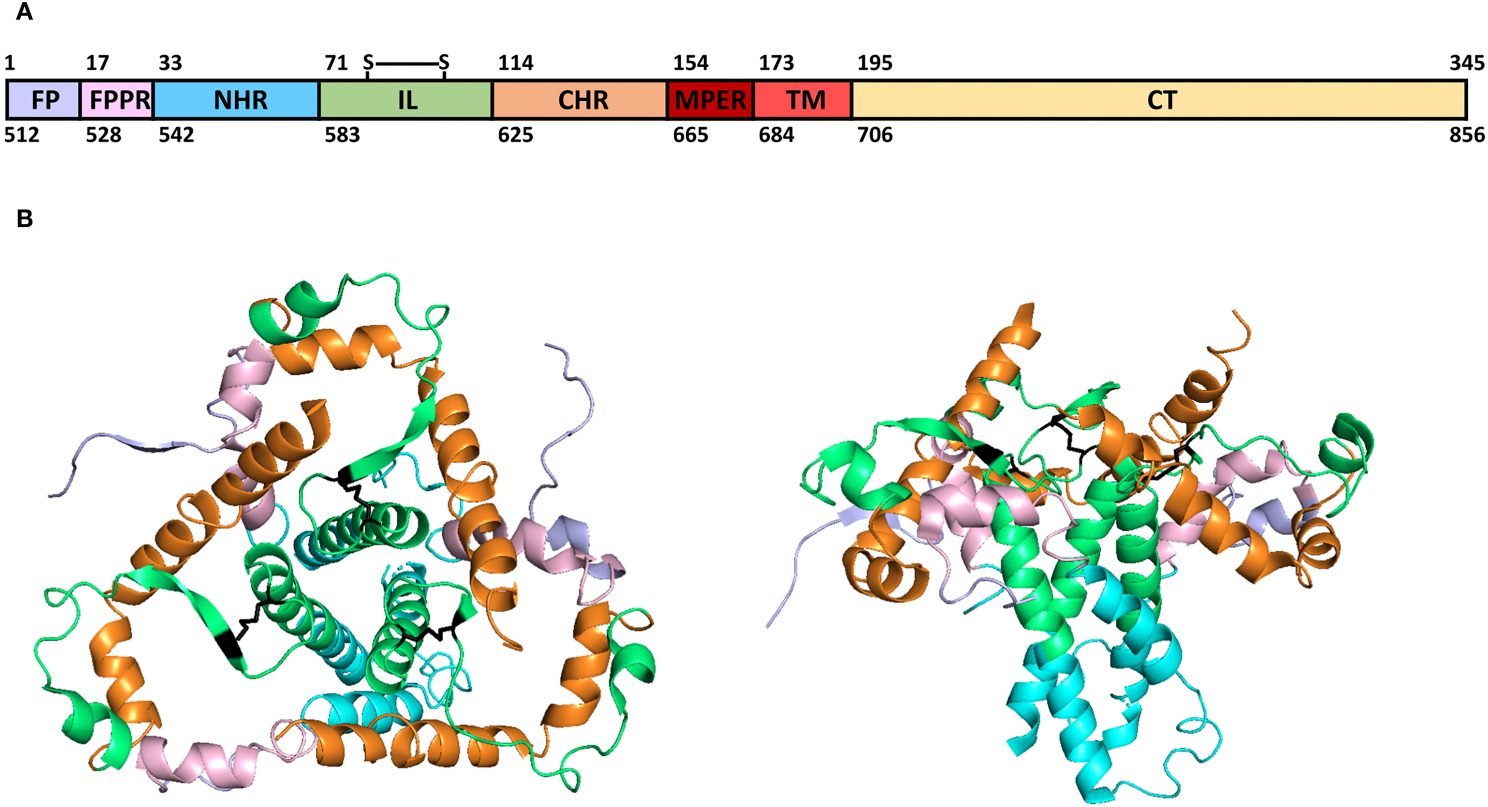
HIV-1 gp41 structural domains **(A)** and 3D model structure **(B)**. Color code: light-purple (fusion peptide, FP), light-pink (fusion peptide proximal region, FPPR), blue (N-terminal alpha-helical region, NHR), green (immune-dominant linker, IL), orange (C-terminal heptad repeat region, CHR), dark-red (membrane-proximal external region, MPER), red (transmembrane region, TM), light-yellow (C-terminal domain, CT), and black (disulfide-bridged loop in IL, S-S). **(A)** Over-domains numbering according to HXB2 isolate aa and below-domains numbering according to HXB2 gp160 nucleotides (Genbank Accession number: K03455). **(B)** Gp41 3D model structure (PDB ID: 6OLP) (Berman et al., 2000; Rantalainen and Cottrell, 2019; Torrents de la Peña et al., 2019).

The human immunodeficiency virus (HIV) envelope transmembrane glycoproteins gp41 and gp36 are located on the HIV-1 and HIV-2 virion's membrane forming trimers with gp120 and gp105 glycoproteins, respectively. These proteins mediate the viral fusion with the host cell's membrane, allowing the entry of genetic material and viral proteins into the cells (Blumenthal et al., 2012). Therefore, they are important targets for the development of fusion inhibitors, such as antiretrovirals (ARV) (Qadir and Malik, 2010), antibodies (Abs) (Caillat et al., 2020), and aptamers (Li et al., 2016) used as HIV treatment. HIV-1 gp41, with 345 amino acids (aa), can be segmented into three domains (Figure 1A): one exposed ectodomain (aa 1-172), a transmembrane region (TM, aa 173-194), and an intraviral not exposed C-terminal domain (CT, aa 195-345). The ectodomain is exposed and can be divided further into distinct functional regions important for fusion and virus infectivity: an N-terminal hydrophobic region termed as fusion peptide (FP, aa 1-16), necessary to bind the virus to the cell membrane, followed by an N-terminal alpha-helical region or N-heptad repeat region (NHR, aa 33-70). These domains were linked by a fusion peptide proximal region (FPPR, aa 17-32) rich in polar aa and critical for HIV-1 fusion and infectivity because it stabilizes the envelope trimers (Lu et al., 2019). A loop immune-dominant linker with a disulfide bridge (IL, aa 71-113) links the NHR to a C-heptad repeat region (CHR, aa 114-153) (Figure 1B). A membrane-proximal external region (MPER, aa 154-172), conformationally flexible, connects the CHR to the TM region (Louis et al., 2016). The gp41 NHR domain is the target of ARV, such as enfuvirtide (T-20), the only clinically approved viral fusion inhibitor for the treatment of HIV infection (Lazzarin, 2005; Oldfield et al., 2005), and MPER for immunogens, as it contains epitopes to broadly neutralizing antibodies (bnAbs), such as 2F5, 4E10, Z13, and 10E8 (Los Alamos HIV Molecular Immunology Database, 2021a).

HIV is one of the most genetically diverse pathogens due to its high mutation and recombination rates, large population size, and rapid replication rate (Hemelaar, 2012). The HIV epidemic is the result of two types of viruses: HIV-1 and HIV-2, which are closely related to SIVcpz (Gao et al., 1999) and SIVsm (Gao et al., 1992), respectively. HIV-1 causes most of the HIV infections worldwide and has been divided according to genetic homology into four groups: M (major or main), N (non-M, non-O) (Simon et al., 1998), O (outlier) (De Leys et al., 1990), and P (Plantier et al., 2009). However, the global HIV epidemic is related to group M (Hemelaar et al., 2019), which has been subdivided into 10 subtypes (A–D, F–H, J–L) and eight sub-subtypes (A1, A2, A3, A4, A5, A6, F1, F2) (Robertson et al., 2000; Salminen, 2000; Leitner et al., 2005; Yamaguchi et al., 2020), at least 118 circulating recombinant forms (CRF) (Los Alamos HIV Sequence Database, 2021a) and uncountable unique recombinant forms (URF). HIV-2 has been classified into nine groups (A-I) and two recombinants (CRF01_AB and URF) (Visseaux et al., 2016).

Since the HIV transmembrane glycoprotein is a key target for human bnAbs and anti-HIV drugs, a better knowledge of aa conservation across structural domains and HIV variants can help to identify conserved targets to direct new therapeutic and diagnostic strategies. Furthermore, each HIV variant presents natural polymorphisms and unique aa changes (V-markers) along the viral genome fixed during viral evolution (Arenas et al., 2016), which have not been described to date in each HIV variant, mainly in HIV-1 group M non-B subtypes and recombinants, which are the majority in the pandemic (Hemelaar et al., 2019), and in HIV-2.

We present, for the first time, the most conserved gp41 domains in each HIV-1 variant per structural domain and anti-gp41 antibody binding domains in the largest panel of HIV-1 variants analyzed to date, identifying the V-markers and the consensus transmembrane glycoprotein sequence for each HIV variant (type, group, subtype, sub-subtype, and CRF).

## Materials and Methods

### HIV Transmembrane Glycoprotein Sequences

In October and November of 2020, we downloaded all available gp41 (HIV-1, 345 aa) and gp36 (HIV-2, 350 aa) nucleotides sequences from Los Alamos National Laboratory (LANL) HIV Sequence Database (Los Alamos HIV Sequence Database, 2021b) selecting one sequence per patient and grouping them per HIV variant (types, groups, subtypes, sub-subtypes, and CRF). URF sequences were not included in this study. They were aligned, edited, and translated into aa with the MEGAv6.0 program (Molecular Evolutionary Genetics Analysis: https://www.megasoftware.net/) (Tamura et al., 2013). MUSCLE function (Multiple Sequence Comparison by Log Expectation) (Edgar, 2004) was used for alignments with HXB2 (HIV-1 subtype B, GenBank accession number: K03455) or BEN (HIV-2 subtype A, GenBank accession number: M30502) reference sequences, removing nucleotides insertions. Sequences with stop codons in unusual positions and groups, subtypes, sub-subtypes, and CRF with <3 sequences were excluded from the study, except group P, as it was necessary to establish the HIV-1 aa sequence consensus.

### Gp41/Gp36 aa Conservation and Inferred Consensus Sequences

Using a bioinformatics tool developed in our laboratory (EpiMolBio program), we analyzed the gp41/gp36 aa conservation of HIV variants with at least three available sequences (except group P, with two sequences). We also inferred the aa consensus sequences for gp41 HIV-1/gp36 HIV-2 and each HIV-1/HIV-2 variant, providing the most conserved aa in each residue.

The new EpiMolBio bioinformatics tool reported the percentage of sequences with a conserved aa in each position of any protein, establishing a color code for this study depending on the frequency of each conserved aa in gp41/gp36: white (<90%), light-blue/light-pink (≥90– <100%), and dark blue-green/fuchsia (100% or complete conservation across considered sequences), respectively. We also studied the level of conserved aa per gp41 structural domain in each HIV-1 non-M group, group M, and HIV-1 consensus sequences. For the analysis, we summed the aa conservation percentages (percentages of most conserved aa) of gp41 residues involved in each secondary structural domain and then divided the sum by the total number of residues per domain.

Finally, we used WebLogo (https://weblogo.berkeley.edu/logo.cgi) (Crooks et al., 2004) to generate a figure showing the HIV-1 and HIV-1 group M gp41, as well as the HIV-2 gp36 consensus sequences, including in each protein position the most frequent aa present in the corresponding alignment. The HIV-1 group M gp41 consensus sequence was generated after the alignment of group M variants consensus sequences, the HIV-1 gp41 consensus sequence was generated after the alignment of the HIV-1 groups (M, N, O, P) consensus sequences, and the HIV-2 gp36 consensus sequence was generated after the alignment of the HIV-2 groups A, B, and the CRF01_AB consensus sequences. The aa letters were represented as large as their conservation percentage with a color code according to their side-chain: in black, non-polar aliphatic (glycine, G; alanine, A; valine, V; leucine, L; methionine, M; isoleucine, I); in dark blue-green, aromatic (phenylalanine, F; tyrosine, Y; tryptophan, W); in fuchsia, polar uncharged (serine, S; threonine, T; cysteine, C; proline, P; asparagine, N; glutamine; Q); in light-blue, positively charged (lysine, K; arginine, R; histidine, H); and in light-pink, negatively charged (aspartic acid, D; glutamic acid, E). Deletions were represented by a yellow “X”.

### HIV-1 Monoclonal Human Antibodies Location

We analyzed the overall conservation in each gp41 secondary-structure domain across the four HIV-1 groups and in the HIV-1 consensus sequence. We also identified the aa conservation level in each HIV-1 group and each anti-gp41 human Abs (bnAbs and non-bnAbs) binding domain described in the LANL HIV Immunology Database (Los Alamos HIV Molecular Immunology Database, 2021a), showing lineal epitopes in blue and non-linear epitopes recognized by bnAbs in orange.

### Gp41/Gp36 Natural Polymorphisms and V-Markers Across HIV Variants

We described the gp41/gp36 natural polymorphisms and V-markers in HIV-1 and HIV-2 variants using the EpiMolBio program. To identify the aa changes present in ≥90% sequences in each HIV-1 or HIV-2 variant (natural polymorphisms), we compared all gp41 sequences with the HXB2 isolate, HIV-1 consensus, and HIV-1 group M consensus sequences, and all gp36 sequences with BEN isolate, and the HIV-2 consensus sequence. Among the natural polymorphisms found, we identified the exclusive V-markers of each HIV-1 or HIV-2 variant, not present in any other HIV variant. The color code for that analysis was light-blue (≥90– <100%) and dark blue-green (100%) for HIV-1 and light-pink (≥90– <100%) and fuchsia (100%) for HIV-2.

We also studied the presence of major T-20 resistance mutations according to the 2019 edition of the International Antiviral Society–USA (2019 IAS-USA) drug resistance mutations list (Wensing et al., 2019) among the V-markers found. Moreover, we looked for L44M change due to association with 1.8-fold resistance to T-20 *in vitro* (Mink et al., 2005).

Furthermore, we examined the presence of natural polymorphisms and specific V-markers on four gp41 positions (N160, W161, F162, and W169), described as key for HIV-1 neutralization by 10E8 (Huang et al., 2012; Kwon et al., 2016), which is a highly potent bnAb-recognizing gp41 MPER (epitope NWFDISNWLWYIK, gp41 positions 160-172) (Los Alamos HIV Molecular Immunology Database, 2021b). Other key reasons to study 10E8 bnAbs were that it was not detected by some serological diagnostic tests targeting gp41 (Smith et al., 2021) and was recently used to design new strategies for the development of a more efficient HIV-1 vaccine (Kuchar et al., 2021).

Finally, some gp41 changes (S23P, S23A, T25A, T27A, and I48P) affecting infectivity, gp41 structure or function (Alsahafi et al., 2015; Lu et al., 2019), were also studied across HIV-1 variants.

## Results

### Analyzed Gp41/Gp36 Sequences and Inferred Consensus Sequences

We downloaded all 18,348 HIV transmembrane glycoprotein sequences from the LANL database. Once sequences with stop codons in unusual positions were discarded and after excluding variants with <3 sequences (except HIV-1 group P), a total of 17,078 gp41/gp36 sequences from 108 variants, including types, groups, subtypes, sub-subtypes, and CRF, were finally used in this study: 16,803 gp41 (HIV-1, 105 variants) and 275 gp36 (HIV-2, three variants) (Table 1). Among the HIV-1 gp41 sequences, 99 belonged to non-M groups (N, O, and P) and 16,704 were ascribed to group M (nine subtypes, six sub-subtypes, and 87 CRF). The gp41 sequences from group M sub-subtype A5, subtype F, CRF30_0206, CRF84_A1D, CRF91_01C, CRF94_cpx, CRF97_01B, CRF101_01B, and CRF102_0107 were not available in LANL. Gp36 sequences from groups E, H, and I were also absent. The variants with the highest representation in HIV-1 group M were subtype B (48.5%), subtype C (23.9%), and recombinant CRF01_AE (13.1%). In HIV-2, the most represented group was A (85.5%).

**Table 1:** Gp41/gp36 LANL sequences were analyzed in this study.

**Variants**				**N**°**SEQS**
			N	11
HIV-1	Non-M Groups		O	86
			P*	2
HIV-1	Group M	Subtypes	A	20
			A1	752
			A2	8
			A3	3
			A4	2
			A5	0
			A6	167
			B	8106
			C	3985
			D	183
			F	0
			F1	122
			F2	15
			G	153
			H	11
			J	8
			K	3
			L	3
HIV-1	Group M	CRF	CRF01_AE	2186
			CRF02_AG	291
			CRF03_AB	5
			CRF04_cpx	8
			CRF05_DF	4
			CRF06_cpx	14
			CRF07_BC	126
			CRF08_BC	62
			CRF09_cpx	5
			CRF10_CD	3
			CRF11_cpx	26
			CRF12_BF	18
			CRF13_cpx	10
			CRF14_BG	12
			CRF15_01B	9
			CRF16_A2D	4
			CRF17_BF	6
			CRF18_cpx	5
			CRF19_cpx	5
			CRF20_BG	4
			CRF21_A2D	4
			CRF22_01A1	13
			CRF23_BG	2
			CRF24_BG	4
			CRF25_cpx	5
			CRF26_A5U	5
			CRF27_cpx	4
			CRF28_BF	5
			CRF29_BF	7
			CRF30_0206	0
HIV-1	Group M	CRF	CRF31_BC	3
			CRF32_06A6	3
			CRF33_01B	7
			CRF34_01B	3
			CRF35_AD	21
			CRF36_cpx	3
			CRF37_cpx	4
			CRF38_BF	1
			CRF39_BF	3
			CRF40_BF	4
			CRF41_CD	3
			CRF42_BF	13
			CRF43_02G	5
			CRF44_BF	3
			CRF45_cpx	5
			CRF46_BF	8
			CRF47_BF	3
			CRF48_01B	3
			CRF49_cpx	5
			CRF50_A1D	4
			CRF51_01B	7
			CRF52_01B	3
			CRF53_01B	4
			CRF54_01B	3
			CRF55_01B	9
			CRF56_cpx	4
			CRF57_BC	7
			CRF58_01B	6
			CRF59_01B	8
			CRF60_BC	5
			CRF61_BC	3
			CRF62_BC	3
			CRF63_02A	15
			CRF64_BC	8
			CRF65_cpx	6
			CRF66_BF	3
			CRF67_01B	2
			CRF68_01B	3
			CRF69_01B	7
			CRF70_BF	3
			CRF71_BF	15
			CRF72_BF	5
			CRF73_BG	2
			CRF74_01B	3
			CRF75_BF	3
			CRF76_01B	2
			CRF77_cpx	4
			CRF78_cpx	3
			CRF79_0107	3
			CRF80_0107	2
			CRF81_cpx	2
HIV-1	Group M	CRF	CRF82_cpx	6
			CRF83_cpx	11
			CRF84_A1D	0
			CRF85_BC	11
			CRF86_BC	3
			CRF87_cpx	3
			CRF88_BC	3
			CRF89_BF	3
			CRF90_BF1	6
			CRF91_01C	0
			CRF92_C2U	5
			CRF93_cpx	3
			CRF94_cpx	0
			CRF95_02B	5
			CRF96_cpx	3
			CRF97_01B	0
			CRF98_06B	1
			CRF99_BF	21
			CRF100_01C	3
			CRF101_01B	0
			CRF102_0107	0
			CRF103_01B	4
HIV-2	Groups		A	235
			B	34
			C	1
			D	1
			E	0
			F	2
			G	1
			H	0
			I	0
HIV-2	CRF		CRF01_AB	6

*In red, variants with <3 sequences. Only HIV-1 group P, with 2 sequences, was included in the study (with an asterisk). N°, number; SEQS, sequences; CRF, circulating recombinant forms*.

**Figure 2:**
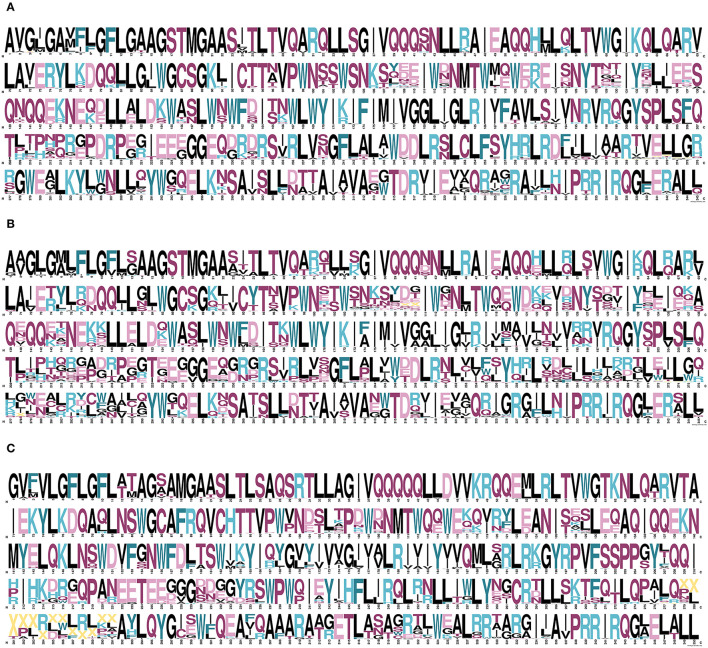
Inferred consensus transmembrane sequences for HIV-1 group M **(A)**, HIV-1 **(B)**, and HIV-2 **(C)**. Each residue includes the most frequent aa present in the corresponding alignment. The aa code letters were represented in proportion to their percentage of conservation. Color code according to their side-chain: non-polar aliphatic (glycine, G; alanine, A; valine, V; leucine, L; methionine, M; isoleucine, I) in black; aromatic (phenylalanine, F; tyrosine, Y; tryptophan, W) in dark blue-green; polar uncharged (serine, S; threonine, T; cysteine, C; proline, P; asparagine, N; glutamine, Q) in fuchsia; positively charged (lysine, K; arginine, R; histidine, H) in light-blue, and negatively charged (aspartic acid, D; glutamic acid, E) in light-pink. Deletions were represented by “X” in yellow.

Consensus sequences at aa level were inferred by EpiMolBio for HIV-1 group M (Figure 2A), HIV-1 (Figure 2B), and HIV-2 (Figure 2C) to study the homology at aa level across variants. The gp41 HIV-1 consensus sequence was generated after aligning the four HIV-1 groups (M, N, O, P) consensus sequences and the HIV-1 group M consensus after aligning 102 group M variants with at least three sequences. The gp36 HIV-2 consensus sequence was inferred after aligning the 275 gp36 LANL sequences ascribed to groups A, B, and CRF01_AB. Supplementary Tables 1, 2 show the inferred HIV-1 and HIV-2 transmembrane consensus sequences in this study, respectively.

### Amino Acid Conservation of Gp41 and Gp36 Across HIV Variants

We identified the gp41 and gp36 residues with ≥90% and 100% conservation across sequences from each analyzed variant (Table 2). The gp41 HIV-1 consensus aa sequence had 38.3% of the 345 gp41 residues conserved in ≥90% of gp41 sequences, and 9% of aa were 100% conserved. Higher conservation of gp36 HIV-2 aa consensus sequence was observed compared to HIV-1 gp41 aa consensus sequence, with 59.7% of 350 gp36 residues conserved in ≥90% of gp36 sequences, and 12.6% of aa totally conserved. Thus, 4 out of 10 aa positions were ≥90% conserved in the HIV-1 gp41 consensus sequence, rising to 6 out of 10 in the HIV-2 gp36 consensus sequence.

**Table 2:** Number and percentage of highly (≥90%, 100%) conserved aa per HIV-1/HIV-2 variant.

					**Conservation**			
					**≥90%**		**100%**	
**Variants**				**SEQS**	**N**°**AA**	**%**	**N**°**AA**	**%**
HIV-1	HIV-1 Consensus			4	132	38.3	31	9
			N	11	296	85.8	252	73
	Non-M Groups		O	86	215	62.3	116	33.6
			P	2	306	88.7	306	88.7
HIV-1	Group M Consensus			102	208	60.3	26	7.5
	Group M	Subtypes	A	20	272	78.8	188	54.5
			A1	752	241	69.9	60	17.4
			A2	8	236	68.4	236	68.4
			A3	3	296	85.8	296	85.8
			A6	167	287	83.2	112	32.5
			B	8106	247	71.6	22	6.4
			C	3985	240	69.6	11	3.2
			D	183	257	74.5	58	16.8
			F1	122	263	76.2	95	27.5
			F2	15	241	69.9	199	57.7
			G	153	247	71.6	90	26.1
			H	11	274	79.4	204	59.1
			J	8	241	69.9	241	69.9
			K	3	289	83.8	289	83.8
			L	3	276	80	276	80
HIV-1	Group M	CRF	CRF01_AE	2186	260	75.4	43	12.5
			CRF02_AG	291	260	75.4	66	19.1
			CRF03_AB	5	311	90.1	311	90.1
			CRF04_cpx	8	249	72.2	249	72.2
			CRF05_DF	4	270	78.3	270	78.3
			CRF06_cpx	14	255	73.9	204	59.1
			CRF07_BC	126	286	82.9	142	41.2
			CRF08_BC	62	281	81.4	173	50.1
			CRF09_cpx	5	290	84.1	290	84.1
			CRF10_CD	3	291	84.3	291	84.3
			CRF11_cpx	26	246	71.3	170	49.3
			CRF12_BF	18	245	71	185	53.6
			CRF13_cpx	10	285	82.6	236	68.4
			CRF14_BG	12	306	88.7	268	77.7
			CRF15_01B	9	243	70.4	243	70.4
			CRF16_A2D	4	273	79.1	273	79.1
			CRF17_BF	6	246	71.3	246	71.3
			CRF18_cpx	5	270	78.3	270	78.3
			CRF19_cpx	5	271	78.6	271	78.6
			CRF20_BG	4	291	84.3	291	84.3
			CRF21_A2D	4	272	78.8	272	78.8
			CRF22_01A1	13	266	77.1	202	58.6
			CRF24_BG	4	299	86.7	299	86.7
			CRF25_cpx	5	270	78.3	270	78.3
			CRF26_A5U	5	257	74.5	257	74.5
			CRF27_cpx	4	254	73.6	254	73.6
			CRF28_BF	5	268	77.7	268	77.7
			CRF29_BF	7	236	68.4	236	68.4
			CRF31_BC	3	291	84.3	291	84.3
			CRF32_06A6	3	321	93	321	93
			CRF33_01B	7	266	77.1	266	77.1
			CRF34_01B	3	327	94.8	327	94.8
			CRF35_AD	21	289	83.8	219	63.5
			CRF36_cpx	3	304	88.1	304	88.1
			CRF37_cpx	4	277	80.3	277	80.3
			CRF39_BF	3	283	82	283	82
			CRF40_BF	4	276	80	276	80
			CRF41_CD	3	341	98.8	341	98.8
			CRF42_BF	13	322	93.3	301	87.2
			CRF43_02G	5	277	80.3	277	80.3
			CRF44_BF	3	286	82.9	286	82.9
			CRF45_cpx	5	265	76.8	265	76.8
			CRF46_BF	8	221	64.1	221	64.1
			CRF47_BF	3	295	85.5	295	85.5
			CRF48_01B	3	299	86.7	299	86.7
			CRF49_cpx	5	268	77.7	268	77.7
			CRF50_A1D	4	298	86.4	298	86.4
			CRF51_01B	7	286	82.9	286	82.9
			CRF52_01B	3	302	87.5	302	87.5
			CRF53_01B	4	288	83.5	288	83.5
			CRF54_01B	3	310	89.9	310	89.9
			CRF55_01B	9	282	81.7	282	81.7
			CRF56_cpx	4	338	98	338	98
			CRF57_BC	7	247	71.6	247	71.6
			CRF58_01B	6	294	85.2	294	85.2
			CRF59_01B	8	295	85.5	295	85.5
			CRF60_BC	5	288	83.5	288	83.5
			CRF61_BC	3	330	95.7	330	95.7
			CRF62_BC	3	322	93.3	322	93.3
			CRF63_02A	15	302	87.5	259	75.1
			CRF64_BC	8	252	73	252	73
			CRF65_cpx	6	294	85.2	294	85.2
			CRF66_BF	3	282	81.7	282	81.7
			CRF68_01B	3	320	92.8	320	92.8
			CRF69_01B	7	280	81.2	280	81.2
			CRF70_BF	3	286	82.9	286	82.9
			CRF71_BF	15	253	73.3	212	61.4
			CRF72_BF	5	257	74.5	257	74.5
			CRF74_01B	3	300	87	300	87
			CRF75_BF	3	316	91.6	316	91.6
			CRF77_cpx	4	304	88.1	304	88.1
			CRF78_cpx	3	279	80.9	279	80.9
			CRF79_0107	3	318	92.2	318	92.2
			CRF82_cpx	6	299	86.7	299	86.7
			CRF83_cpx	11	319	92.5	298	86.4
HIV-1	Group M	CRF	CRF85_BC	11	308	89.3	251	72.8
			CRF86_BC	3	305	88.4	305	88.4
			CRF87_cpx	3	312	90.4	312	90.4
			CRF88_BC	3	303	87.8	303	87.8
			CRF89_BF	3	290	84.1	290	84.1
			CRF90_BF1	6	258	74.8	258	74.8
			CRF92_C2U	5	234	67.8	234	67.8
			CRF93_cpx	3	277	80.3	277	80.3
			CRF95_02B	5	294	85.2	294	85.2
			CRF96_cpx	3	305	88.4	305	88.4
			CRF100_01C	3	293	84.9	293	84.9
			CRF103_01B	4	302	87.5	302	87.5
HIV-2	HIV-2 Consensus			3	209	59.7	44	12.6
	Groups		A	235	231	66	55	15.7
			B	34	251	71.7	159	45.4
	CRF		CRF01_AB	6	290	82.9	290	82.9

*Data showed the number of aa conserved in ≥90 or 100% of sequences per analyzed variant. Gp41 (HIV-1), 345 aa; gp36 (HIV-2), 350 aa. Gp41 HIV-1 consensus sequence was generated after the alignment of the four HIV-1 groups (M, N, O, P) consensus sequences, the HIV-1 group M consensus after the alignment of 102 group M variants with ≥3 sequences, and the HIV-2 consensus after the alignment of three HIV-2 variants. N°, number; SEQS, sequences; AA, amino acids; %, aa percentage; CRF, circulating recombinant forms*.

For HIV-1 groups, the highest percentage of conserved aa in ≥90%/100% of their HIV-1 gp41 LANL sequences were found in groups P (88.7%/88.7%) and N (85.8%/73%), followed by O (62.3%/33.6%) and M (60.3%/7.5%). Therefore, group P was the HIV-1 non-M group with the highest number of highly conserved gp41 residues, while group O had the fewest.

Among the 16,704 group M gp41 sequences, CRF46_BF had the lowest number of aa present in ≥90% of sequences (64.1%, eight sequences) and CRF41_CD the highest (98.8%, three sequences). When considering the completely conserved (100%) residues, subtype C showed the lowest number (3.2%, 3,985 sequences) and CRF41_CD the highest (98.8%, three sequences). Thus, among group M variants, CRF41_CD was the most conserved variant, while subtype C was the least conserved.

Regarding HIV-2, the variant with the highest percentage of ≥90%/100% conserved aa in gp36 protein was CRF01_AB (82.9%/82.9%), followed by group B (71.7%/45.4%) and group A (66%/15.7%).

### Gp41 Conservation in Each HIV-1 Group per Structural Domain and Anti-Gp41 Antibody Binding Sites

When we studied the level of conserved aa in each gp41 structural domain, we observed that gp41 conservation differed between HIV-1 groups and structural domains (Table 3), ranging from 66.4% to 98.9% conservation. The gp41 domain in the HIV-1 consensus sequence with the highest conservation was NHR (86.2%), followed by FPPR (84.9%), MPER (84.2%), and FP (84%). We observed a high conservation percentage in FPPR (96%) and NHR (95.4%) domains in the group M consensus sequence, despite the lower overall gp41 conservation *vs*. the other three HIV-1 groups. Group N gp41 presented the most conserved FP (98.9%) and FPPR (98.8%) domains, group O the best conserved MPER domain (95.7%), and group P the most conserved NHR (98.7%), TM (95.5%), IL (95.3%), and CT (93.7%) gp41 domains (Table 3). CHR domain presented the highest conservation (95.0%) in groups N and P. Considering all analyzed HIV-1 variants, the FP, FPPR, NHR, and MPER were the gp41 domains with the highest (≥84%) number of conserved aa, being the target for most anti-gp41 Abs. The least conserved domains in the HIV-1 consensus sequence were TM (69%) and CT (66.4%) located inside the virus.

**Table 3:** Percentage of aa conservation in each gp41 structural domain across HIV-1 groups and HIV-1 consensus sequences.

	**% AA Conservation HIV-1 GP41**								
**Gp41 structural domains**	**FP**	**FPPR**	**NHR**	**IL**	**CHR**	**MPER**	**TM**	**CT**	**GP41**
Gp41 residues	1–16	17–32	33–70	71–113	114–153	154–172	173–194	195–345	1–345
Group N	**98.9**	98.8	96.5	95.0	95.0	90.9	94.6	93.5	94.6
Group O	**97.7**	87.6	95.3	79.2	83.8	95.7	89.0	86.0	87.3
Group P	93.8	90.6	**98.7**	95.3	95.0	89.5	95.5	93.7	94.3
Cons. Group M	86.7	**96.0**	95.4	86.6	79.6	86.1	91.9	81.0	85.1
Cons. HIV-1	84.0	84.9	**86.2**	73.3	70.7	84.2	69.0	66.4	72.8

*For the analysis, we used the aa conservation percentages per residue (percentages of most conserved aa) of each HIV-1 group and HIV-1 consensus sequence provided in Supplementary Table 1 with color code. After identifying the residues of each secondary structural domain, the sum of the aa conservation percentages of each of them was divided by the total number of residues per domain. In color, the highest conservation level in each structural domain. In bold, the most conserved structural domain in each HIV-1 group and HIV-1 consensus sequence. Gp41 residues according to HXB2 HIV-1 isolate. N°, number; AA, amino acids; Cons, aa consensus sequence; FP, fusion peptide; FPPR, fusion peptide proximal region; NHR, N-terminal alpha-helical region; IL, immune-dominant linker; CHR, C-terminal heptad repeat region; MPER, membrane-proximal external region; TM, transmembrane region; CT, C-terminal domain*.

**Figure 3:**
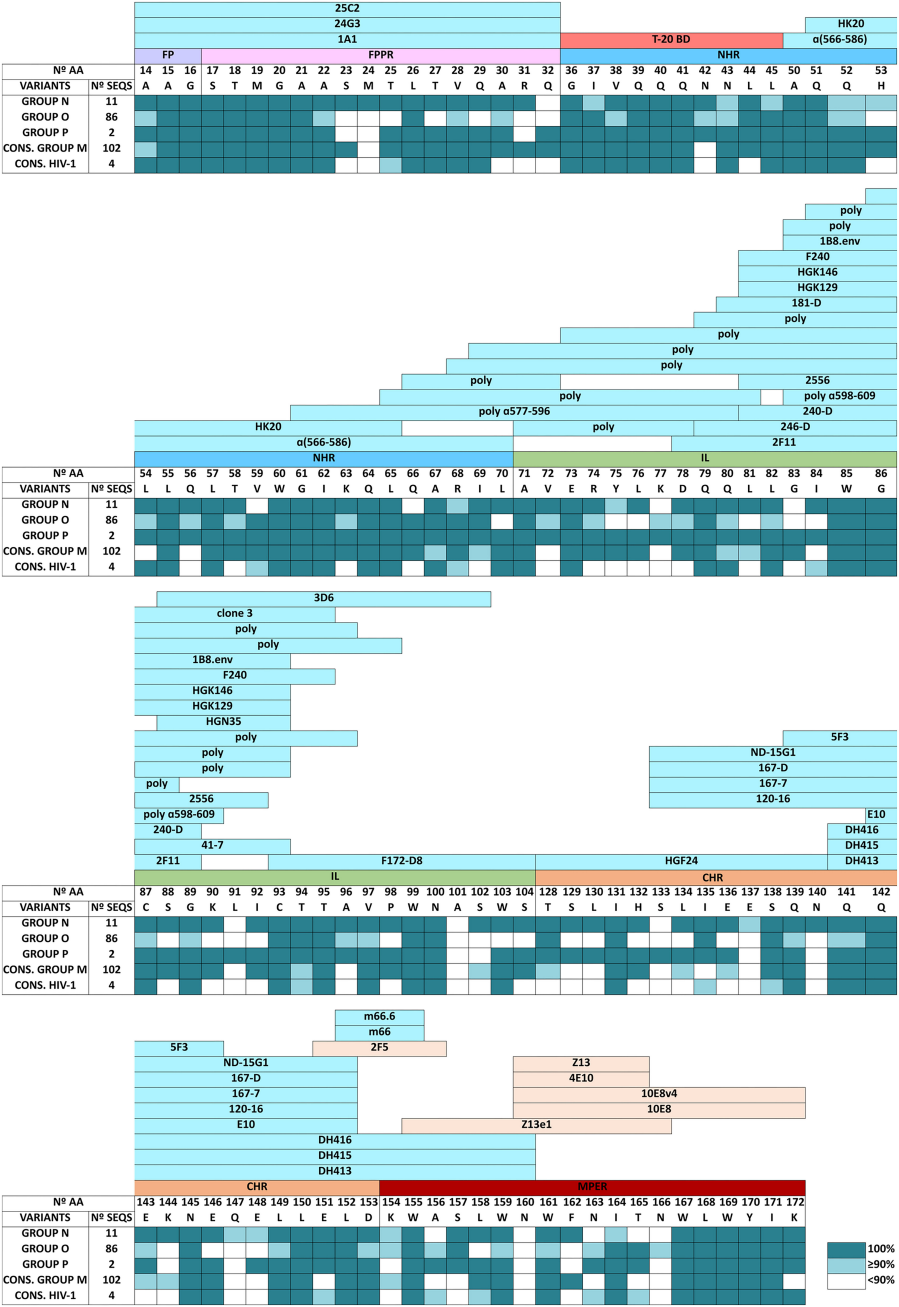
Amino acid conservation level in gp41 residues involved in anti-gp41 human antibody binding domains described in LANL HIV Immunology Database across HIV-1 groups and HIV-1 consensus sequences. Data showed the percentage of the most conserved aa with the following conservation color code: white (aa conserved <90%), light-blue (aa conserved ≥90%– <100%), and dark blue-green (aa conserved 100%). Anti-gp41 human antibodies include bnAbs and non-bnAbs described in Los Alamos HIV Immune Database (Los Alamos HIV Molecular Immunology Database, 2021a). Linear epitopes are shown in blue and non-linear epitopes, recognized by broadly neutralizing antibodies (bnAbs), are shown in orange. We also indicated the T-20 fusion inhibitor binding domain in red (T-20BD). N°, number; AA, amino acids; SEQS, sequences; Cons, aa consensus sequence; FP, fusion peptide; FPPR, fusion peptide proximal region; NHR, N-terminal alpha-helical region; IL, immune-dominant linker; CHR, C-terminal heptad repeat region; MPER, membrane-proximal external region; TM, transmembrane region; CT, C-terminal domain; G, glycine; A, alanine; V, valine; L, leucine; M, methionine; I, isoleucine; F, phenylalanine; Y, tyrosine; W, tryptophan; S, serine; T, threonine; C, cysteine; P, proline; N, asparagine; Q, glutamine; K, lysine; R, arginine; H, histidine; D, aspartic acid; E, glutamic acid; poly, polyclonal.

The conservation level in each domain for human anti-gp41 bnAbs and non-bnAbs across HIV-1 groups was also described (Figure 3), located between the FP and MPER domains since TM and CT (the less conserved gp41 domains according to our data) are not exposed. The bnAbs are directed to CHR (2F5) and mainly to the MPER domain (2F5, Z13, 4E10, 10E8, and derivatives). No anti-gp41 Ab-binding sites were shown in LANL in the first 13 aa of the FP domain, in the 17 first positions of NHR, in the last 9 residues of IL, and the first 14 aa of CHR. The same was true in the target peptide to the fusion inhibitor T-20 (GIVQQQNNLL, NHR, residues 36-45), even though it presented high conservation across HIV-1 groups (Figure 3).

### Natural Polymorphisms and V-Markers

**Figure 4:**
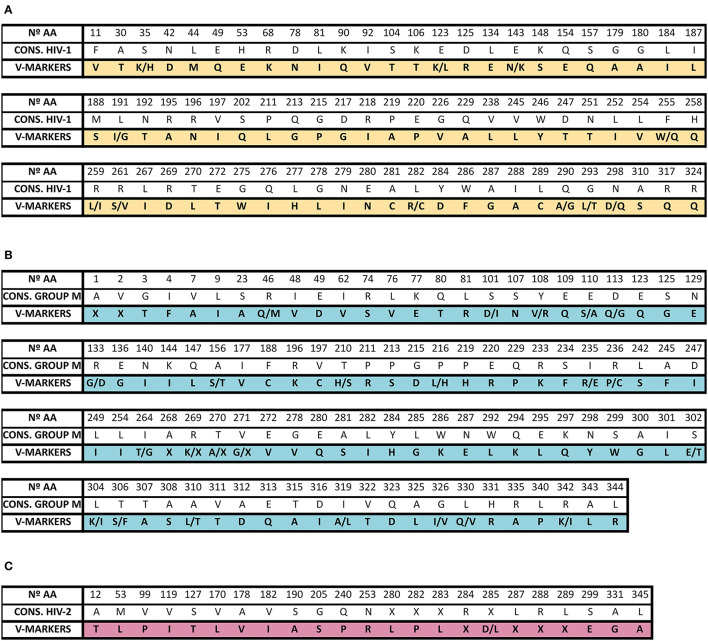
Location of exclusive transmembrane glycoprotein V-markers in non-M groups **(A)**, HIV-1 group M variants **(B)**, and HIV-2 variants **(C)**. V-markers in gp41 HIV-1 non-M groups are described with respect to the HIV-1 consensus sequence (yellow color). V-markers in gp41 HIV-1 group M variants are described with respect to the HIV-1 group M consensus sequence (light-blue color). V-markers in gp36 HIV-2 variants are described with respect to the HIV-2 consensus sequence (light-pink color). These V-markers and their corresponding variant can be also found in Supplementary Table 3 (for HIV-1) and Supplementary Table 4 (for HIV-2). V-markers, aa changes that were specific for each variant present in ≥90% of gp41 or gp36 sequences of an exclusive HIV-1 or HIV-2 variant; N°, number; AA, amino acids; CONS, aa consensus sequence; G, glycine; A, alanine; V, valine; L, leucine; M, methionine; I, isoleucine; F, phenylalanine; Y, tyrosine; W, tryptophan; S, serine; T, threonine; C, cysteine; P, proline; N, asparagine; Q, glutamine; K, lysine; R, arginine; H, histidine; D, aspartic acid; E, glutamic acid; X, aa deletion.

All-natural polymorphisms (aa present in ≥90% sequences) found in each HIV variant are shown in Supplementary Table 3 (HIV-1) and Supplementary Table 4 (HIV-2). The number of polymorphisms in gp41 across HIV-1 non-B variants (different from subtype B) increased when using the HXB2 subtype B sequence as reference. HIV-2 group A presented fewer polymorphisms in gp36 when HIV-2 BEN subtype A group was used as reference. Figure 4 shows the natural polymorphisms that could be considered as exclusive V-markers for HIV-1 gp41 non-M groups (86 V-markers, Figure 4A), for HIV-1 group M gp41 variants (120 V-markers, Figure 4B), and HIV-2 gp36 variants (24 V-markers, Figure 4C). The specific V-markers per variant can be found in Supplementary Tables 3, 4.

No V-markers associated with T-20 major resistance were found in HIV-1 groups N, O, P, group M, and HIV-2 variants. Only M44 was present in the group P consensus gp41 sequence (Supplementary Tables 1, 3). Group O gp41 consensus sequence carried D42 V-marker, but no T42, a residue associated with high T-20 resistance.

After the analysis of the four gp41 key positions (N160, W161, F162, and W169) for HIV-1 neutralization by the anti-MPER bnAb 10E8, we observed high (90–100%) conservation of W161 and W169 in the transmembrane consensus sequences across HIV-1 variants. L162 appeared in group O and L/M162 in group P gp41 consensus sequence. Residue 160 showed the highest variability, carrying the group P and many HIV-1 group M variants another aa, mainly serine.

Finally, regarding changes in gp41 residues (A23, P23, A25, A27, and P48) affecting infectivity, gp41 structure, or function, we observed that A23 appeared in groups P and O and in CRF63_02A gp41 consensus sequences, while A25 appeared in group O, subtype L, CRF41_CD, and CRF47_BF group M variants. V48 appeared in the CRF90_BF1 gp41 consensus sequence but P48 did not.

## Discussion

This is the most up-to-date descriptive study related to HIV-1/HIV-2 transmembrane envelope proteins, providing the conservation level and the V-markers in each HIV variant, identifying the conserved gp41 domains in each HIV-1 group per structural domain and per anti-gp41 antibody binding site in the most extensive panel of HIV-1 gp41/HIV-2 gp36 sequences (*n* = 17,078) and variants (105 HIV-1 variants, three HIV-2 variants) analyzed to date. Our in-house bioinformatics tool was also used to establish consensus sequences for gp41/gp36 proteins to study the aa level conservation across HIV variants.

The results have shown that the degree of conservation of the protein can differ across HIV variants and transmembrane structural domains, as our group previously described with fewer sequences and variants (Holguín et al., 2007). The higher level of conserved residues across variants in gp36 (275 sequences) *vs*. gp41 (16,803 sequences) could be explained by the lower number of gp36 sequences in LANL due to the lower HIV-2 prevalence and worldwide infections (1–2 of 38 million HIV infections) explained by its lower transmissibility and virulence *vs*. HIV-1 (Azevedo-Pereira and Santos-Costa, 2016; Clinical Info and gov, 2019; Kapoor and Padival, 2021). A higher level of fully conserved aa observed in HIV-1 non-M groups (99 sequences) *vs*. group M (16,704 sequences) could also be explained by the lower prevalence of groups N, O, and P in the pandemic (Mourez et al., 2013). The same happens in most CRF, with few available gp41 sequences in LANL, due to their low prevalence and the absence of gp41 sequencing in countries where they circulate, with sequencing or research not always available.

All gp41 secondary structure ectodomains presented >70% of conservation (HIV-1 consensus sequence), which supports their key role in the viral cycle and the importance of structure maintenance for virus-cell membranes fusion and viral entry (Pancera et al., 2014). Our data revealed that the best-conserved gp41 domains were N-HR (86.2%), FPPR (84.9%), MPER (84.2%), and FP (84%). It is essential to highlight that precisely NHR, FP, and FPPR gp41 domains have recently been implicated in the interaction with the fusion inhibitor T-20, which prevents the virus from entering the cell (Xu et al., 2019).

Regarding the gp41-CT region (aa 195-345), located inside the virus, it was the least conserved domain in the HIV-1 and group M gp41 consensus sequences, despite its important role in gp41 structure and function (Fernandez and Freed, 2018). CT domain is involved in envelope conformation (Castillo-Menendez et al., 2018), being essential for efficient envelope incorporation into budding HIV-1 particles (Murakami and Freed, 2000), and requiring interaction with gag matrix protein (MA) (Wyma et al., 2000; Eastep et al., 2021). However, the domains in each protein involved in this interaction are still unknown (Fernandez and Freed, 2018). A link between the matrix trimers' formation and the binding between MA and gp41 CT has also been reported (Alfadhli et al., 2016).

The critical role of the FPPR or polar region at the N terminus of gp41 for HIV-1 fusion and infectivity by stabilizing envelope trimers (Lu et al., 2019) could explain the high gp41 aa conservation percentage found in this domain. S23 within FPPR is structurally essential for maintaining HIV-1 envelope trimer, viral fusogenicity, and infectivity (Lu et al., 2019). Single or combined mutations S23P, T25A, and T27A in the FPPR region abolished or significantly decreased HIV-1 infectivity without affecting viral production, and S23A change significantly reduced HIV-1 infectivity and fusogenicity but not envelope expression and cleavage (Lu et al., 2019). In our study, only S23A and T25A substitutions appeared in a low number of variants with low prevalence in the pandemic. The absence of I48P gp41 change in our sequence set could be explained by its high impact on the quaternary conformation and function of the envelope glycoprotein trimer (Alsahafi et al., 2015).

Analyzing the genetic variability of HIV glycoprotein transmembrane within its immunodominant epitopes is important for understanding its possible impact on HIV Abs detection (Dorn et al., 2000; Dong et al., 2005; Smith et al., 2021). HIV-1 bnAbs can neutralize most HIV-1 strains from diverse genetic and geographic backgrounds (Binley et al., 2004; Wang and Zhang, 2020). The anti-gp41 bnAbs can recognize the MPER (Huang et al., 2012) and FP domains (Yuan et al., 2019), as well as the gp120/gp41 interphase (Huang et al., 2014; Scharf et al., 2014; Wang and Zhang, 2020). The conformational plasticity of FP could facilitate the recognition of the virus by bnAbs (Yuan et al., 2019). It is also known that HIV-1 variability can impact on bnAbs reactivity in HIV diagnostic tests targeting gp41, leading to non-reactive results with different serological diagnostic assays (Smith et al., 2021). Furthermore, the identification of HIV antibody binding domains is critical for vaccine development studies (Kuchar et al., 2021). Many bnAbs directed to gp41 have been described (Los Alamos HIV Molecular Immunology Database, 2021a), and the identification of the aa conservation level across HIV variants on the recognized epitopes by each bnAb and in those key gp41 residues for viral neutralization is of particular interest. The high variability found in key gp41 residue 160 across some HIV-1 variants could explain the previously reported failure of 10E8 recognition by some different serological diagnostic tests (Smith et al., 2021). We could not analyze the gp41 aa conservation in the gp41 target sequence per each failing diagnostic test because manufacturers do not provide detailed information regarding which part of the gp41 sequence was targeted in their HIV diagnostic assays detecting the transmembrane protein, which can also differ across assays. The provided information in the Supplementary Tables of the manuscript can help manufacturers and other researchers design new gp41-based molecular and serological diagnostic tests to identify those HIV-1 variants whose diagnosis could be compromised by viral genetic variability.

Natural infection by HIV-2 also leads to the elicitation of high titers of bnAbs against primary HIV-2 strains (De Silva et al., 2012; Kong et al., 2012; Özkaya Sahin et al., 2012), although not all bnAbs to HIV-2 neutralize HIV-1 variants (Björling et al., 1993). In fact, MPER-specific Abs induced by vaccination with recombinant gp36 proteins in rats did not neutralize HIV-2 (Behrendt et al., 2012). It is known that HIV types present different mechanisms for the processing of envelope glycoproteins from a smaller env precursor in HIV-2 (gp140) than in HIV-1 (gp160) (Rey et al., 1989). To date, unfortunately, the exact aa residues of each secondary structure in HIV-2 gp36 have not been specified. However, some conserved gp36 epitopes have been reported (Jadhav et al., 2011), as well as immunogenic sites, antibody binding sites in the TM and IL region of HIV-2 transmembrane gp36 (Chiodi et al., 1993), and other epitopes recognized by bnAbs in HIV-2 envelope gp140 (Kong et al., 2012).

Previous studies showed the importance of consensus sequence establishment to guide vaccine development (Ellenberger et al., 2002; Sliepen et al., 2019). For the first time, our study also provides the aa consensus sequence of the transmembrane glycoprotein in each HIV variant (type, group, subtype, sub-subtype, and CRF). Moreover, we showed the conservation level across their sequences, which could be helpful to look for highly conserved peptides to direct new ARV, Abs, aptamers, probes, or primers to control or diagnose HIV infection regardless of the HIV variant. Furthermore, we showed the first identification of specific natural polymorphisms of gp41 and gp36 that can be considered as V-markers for all HIV-1 and HIV-2 variants, which should be considered in the new strategies for developing HIV-1 vaccines based on epitopes recognized by bnAbs (Kuchar et al., 2021). The exclusive HIV V-markers identified in gp41/gp36 sequences could help in faster and preliminary HIV variant identification if required, before doing the phylogenetic study, the gold standard method for correct HIV variant characterization. New studies are required to evaluate the structural and biological impact of the different levels of aa conservation in gp41 across HIV-1 variants and the specific V-markers found in the viral transmembrane protein.

Long-term exposure to the first entry inhibitor T-20 induces drug-resistant mutations (Pérez-Alvarez et al., 2006). Interestingly, none of the variants had V-markers associated with major resistance to T-20, as we previously reported testing 79 different HIV variants from naïve patients (Holguín et al., 2007). Thus, no natural major resistance mutations to T-20 were observed. The L44M change found in group P consensus sequence, previously associated with 1.8-fold resistance to T-20 *in vitro* (Mink et al., 2005), was previously found in T-20 naïve subjects from China (Chang et al., 2021), maybe reflecting a resistance transmission during primoinfection. New HIV-1 fusion inhibitors are under development (Luque and Camarasa, 2021) and previous studies showed that optimized T-20 derivates could have been effective inhibitors of infection for multiple HIV-1 variants (Chen et al., 2019).

The main limitation of the study was the absence of LANL sequences from some HIV-1 (sub-subtype A5, subtype F, CRF30_0206, CRF84_A1D, CRF91_01C, CRF94_cpx, CRF97_01B, CRF101_01B, CRF102_0107) and HIV-2 variants (groups E, H, I), as well as the scarce number of HIV gp41/gp36 sequences in other 14 HIV variants with <3 sequences in LANL, which meant that they could not be included in the analysis (except group P).

The information provided in this manuscript aims to help other researchers studying the biological, therapeutical, diagnostic, or structural role of gp41 to identify the natural polymorphisms and specific V-markers per variant in each gp41/gp36 residue or epitope according to their interest. This study will also be useful for a more rational design of anti-gp41 drugs and vaccines and future HIV molecular diagnostic tests directed to transmembrane HIV protein.

## Data Availability Statement

The original contributions presented in the study are included in the article/Supplementary Files, further inquiries can be directed to the corresponding authors.

## Author Contributions

AV-A downloaded and analyzed the HIV LANL sequences under study, validated some EpiMolBio functions necessary for sequences analyses, performed the computations, discussed results, and wrote the first draft of the manuscript. RR developed the in-house EpiMolBio bioinformatics program, validated some EpiMolBio functions necessary for sequences analyses, discussed results, and reviewed the final version of the manuscript. ÁH designed and supervised the study, reviewed and edited the manuscript, funding application, and project administration. All authors approved the final version submitted.

## Funding

This research and AV-A's contract were funded by Instituto de Salud Carlos III (PI18/00904 Plan Estatal de Investigación Científica y Técnica y de Innovación 2013-2016) and co-financed by the European Regional Development Fund “A way to achieve Europe” (ERDF). RR was funded by FONDOS FUR 2020/0285. The funder had no role in the design of the study, in the collection, analyses, or interpretation of data, in the writing of the manuscript, or in the decision to publish the results.

## Conflict of Interest

The authors declare that the research was conducted in the absence of any commercial or financial relationships that could be construed as a potential conflict of interest.

## Publisher's Note

All claims expressed in this article are solely those of the authors and do not necessarily represent those of their affiliated organizations, or those of the publisher, the editors and the reviewers. Any product that may be evaluated in this article, or claim that may be made by its manufacturer, is not guaranteed or endorsed by the publisher.
